# Alpha-Lipoic Acid Alleviates Lead-Induced Testicular Damage in Roosters by Reducing Oxidative Stress and Modulating Key Pathways

**DOI:** 10.3390/toxics13050341

**Published:** 2025-04-25

**Authors:** Jiahao Sun, Rahmani Mohammad Malyar, Nanwei Ye, Yueyue Wang, Quanwei Wei, Fangxiong Shi, Yansen Li

**Affiliations:** 1 College of Animal Science and Technology, Nanjing Agricultural University, Nanjing 210095, China; 2022205014@stu.njau.edu.cn (J.S.); 2021205039@stu.njau.edu.cn (R.M.M.); 2019205009@njau.edu.cn (N.Y.); 2022105025@stu.njau.edu.cn (Y.W.); weiquanwei@njau.edu.cn (Q.W.); fxshi@njau.edu.cn (F.S.); 2 Veterinary Science Faculty, Nangarhar University, Jalalabad City 2601, Nangarhar, Afghanistan

**Keywords:** Pb, alpha-lipoic acid, testicular toxicity, oxidative stress, rooster

## Abstract

(1) Background: This study aimed to detect whether alpha-lipoic acid (ALA) supplementation could reduce lead (Pb)-induced testicular toxicity in roosters. (2) Methods: A total of 48 roosters, aged 20 weeks, were selected and randomly allocated to six treatment groups: basic diet (CON); CON + 150 mg/kg (CH_3_OO)_2_Pb (LPB); CON + 300 mg/kg (CH_3_OO)_2_Pb (HPB); CON + 300 mg/kg ALA (ALA); LPB + 300 mg/kg ALA (ALP); and HPB + 300 mg/kg ALA (AHP). (3) Results: The testicular Pb content was obviously higher in the LPB and HPB groups than in the CON group, while ALA supplementation reduced the testicular Pb content (*p* < 0.05). Roosters showed a significant increase in serum testosterone, sperm viability, sperm concentration, and testicular score in the AHP group compared with the HPB group. Pb exposure caused a remarkable increase in sperm abnormality and testicular malondialdehyde level, which were down-regulated by ALA supplementation (*p* < 0.05). RNA sequencing identified 227 differentially expressed genes (DEGs) between the HPB and CON groups and 220 DEGs between the HPB and AHP groups. (4) Conclusions: ALA supplementation mitigated Pb-induced testicular damage, suggesting its potential as a therapeutic agent for Pb toxicity in birds and potentially other species.

## 1. Introduction

Lead (Pb) is a widespread heavy metal commonly used in industries such as Pb-acid battery production, automobile repair, paint manufacturing, and smelting [1]. Due to its prolonged biological half-life, Pb persists in the environment, accumulating through the food chain and presenting significant risks to biological health [2]. Chronic Pb exposure can result in its accumulation in organisms, leading to cellular, tissue, and systemic damage. Growing evidence indicates that Pb exposure is associated with testicular damage and male reproductive dysfunction in rat, ram, boar, and bull [3]. Studies have reported reduced testosterone levels, decreased sperm quality, the degradation of spermatogenic tubules, and impaired spermatogenesis in male mice exposed to Pb [4]. Currently, wild birds hold an important position in the ecosystem, and the safety of their breeding is of great concern. However, whether Pb contamination severely impacts the reproductive performance of male birds and the possible mechanisms underlying Pb-induced reproductive damage in birds remain unclear.

Pb-induced testicular damage was characterized by oxidative stress and apoptotic cell death, as demonstrated by previous studies. Oxidative damage occurs when excessive reactive oxygen species (ROS) overwhelm the antioxidant defense system [5]. The testis, a primary target of oxidative stress, is pivotal in the pathogenesis of male infertility [6]. Cellular redox balance was frequently dysregulated in the testes of Pb-exposed rat, leading to decreased antioxidant enzyme levels and increased oxidative stress [7,8]. Excessive ROS accumulation in pathological conditions induced oxidative damage, triggering both apoptosis and autophagy [9]. Alpha-lipoic acid (ALA) is a naturally occurring antioxidant found abundantly in vegetables, such as broccoli and spinach, and in animal tissues, such as the liver, heart, and kidney, as well as in yeast [10,11]. ALA is absorbed and accumulated through dietary intake, and its high lipophilicity allows it to cross biological barriers and membranes [11]. Known for its powerful antioxidant properties, ALA scavenges various free radicals, thus mitigating oxidative stress both in vivo and in vitro [10]. It is widely used to treat reproductive disorders and prevent testicular damage caused by both endogenous and exogenous factors. A previous study demonstrated that ALA enhanced spermatogenesis in aging breeding roosters by improving testicular antioxidant capacity [12]. Additionally, ALA has been shown to alleviate toxic damage in rat testes via chelating cadmium [13]. However, it remains unclear whether ALA can protect against Pb-induced testicular damage in birds.

During the process of Pb-induced tissue damage, molecular processes such as autophagy, inflammatory response, and cell adhesion have also been found to be involved [1,14,15]. However, further confirmation is required regarding which pathway plays a key role in Pb-induced testicular damage in roosters. In commercial poultry farms, the Pb content in rooster feeds shall not exceed 5 mg/kg according to the Hygienical Standard for Feeds of China (GB 13078-2017) [16]. Moreover, technology used to collect rooster semen is commonly adopted in poultry farming, making it an ideal model to detect bird semen quality and study related mechanisms. Thus, this study aimed to investigate whether ALA intervention could mitigate Pb-induced poor semen quality and testicular damage by establishing a combined Pb poisoning and ALA treatment model in roosters. An RNA sequencing analysis was performed to determine the possible key molecular mechanism underlying Pb-induced testicular impairment and the beneficial effects of ALA.

## 2. Materials and Methods

### 2.1. Birds and Treatments

The animal experiments were authorized by the Animal Welfare Committee of Nanjing Agricultural University (SYXK(Su)2021-0086). A total of 48 broiler breeder roosters, aged 20 weeks, were selected for this study. The roosters were randomly allocated to six treatment groups, with eight roosters per group, and each rooster was individually raised in a separate cage with welded wire floor. The six groups were as follows: (1) roosters fed a basic diet (CON); (2) roosters fed a basic diet supplemented with 150 mg/kg (CH_3_OO)_2_Pb (LPB); (3) roosters fed a basic diet supplemented with 300 mg/kg (CH_3_OO)_2_Pb (HPB); (4) roosters fed a basic diet supplemented with 300 mg/kg ALA (ALA); (5) roosters fed a basic diet supplemented with 150 mg/kg (CH_3_OO)_2_Pb and 300 mg/kg ALA (ALP); and (6) roosters fed a basic diet supplemented with 300 mg/kg (CH_3_OO)_2_Pb and 300 mg/kg ALA (AHP). In order to successfully establish a model of chronic Pb poisoning in roosters, the supplement levels and exposure period of (CH_3_OO)_2_Pb were optimized based on previous studies [17,18,19]. The formal experiment lasted for eight weeks. The standard diet used in this experiment was designed according to the nutrient requirements outlined by the National Research Council (1994; Table S1). All birds were housed in a temperature- and light-controlled room with air temperature maintained between 22 and 25 °C under a 13 h light/dark cycle. Each rooster was fed a restricted diet of 125 g per day and had free access to water.

The live body weight of each rooster was recorded before and after the experiment, respectively. Blood samples were collected from the wing vein, and the serum was stored at −20 °C. Following euthanasia by CO_2_ asphyxiation, the testes were weighed and immediately harvested. The left testis was gently washed using phosphate-buffered saline (pH 7.4) and then stored at −80 °C, while the right testis was preserved in Davidson’s Fixative for histological analysis.

### 2.2. Semen Evaluation

In this study, ejaculate volume, sperm concentration, sperm viability, sperm abnormalities, and membrane functional integrity were evaluated. At the end of the 8-week feeding trial, the abdominal massage method was used to collect semen samples of individual roosters, and the parameters were measured immediately. Ejaculate volume was quantified using the graduated collection tubes, while sperm concentration was detected using a Neubauer hemocytometer. Sperm morphological abnormalities were assessed using Diff-Quik staining (G1541, Solarbio, Beijing, China) following the manufacturer’s instructions. Sperm viability was evaluated through eosin-nigrosin smears, where unstained (viable) and stained (dead) sperm were counted after incubation with the eosin-nigrosin solution.

Sperm membrane functional integrity was measured using a hyperosmotic swelling test. Briefly, semen samples were diluted in a hypoosmotic solution with an osmolarity of 75 milliosmoles per kg. The solution consisted of 16.3 mM trisodium citrate and 48.4 mM D-fructose. Subsequently, the diluted samples were incubated at 37 °C for 30 min. Spermatozoa exhibiting a curled tail was considered to have a functionally intact plasma membrane. All sperm slides were evaluated under a phase-contrast microscope (Lx 400, Nikon, Tokyo, Japan). On each slide, five different fields of view were examined, and at least 200 spermatozoa were counted.

### 2.3. Pb Content Analysis

In quartz tubes, 0.2 g of the testis was digested using 4.0 mL of concentrated HNO_3_ and 1.0 mL of H_2_O_2_. The Pb content was measured using inductively coupled plasma mass spectrometry (ICP-MS) (Agilent, Santa Clara, CA, USA).

### 2.4. Determination of Serum Testosterone and Testicular Oxidative Status

A commercial enzyme-linked immunosorbent assay (ELISA) kit (Mlbio, Shanghai, China) was applied to determine serum testosterone of roosters in each group. A microplate reader (Thermo Scientific, Sunnyvale, CA, USA) with an analysis range of 25–800 pg/mL and an assay sensitivity of 1 pg/mL was used to measure absorbance at 450 nm. The supernatants of testicular homogenates were prepared for the determination of malondialdehyde (MDA) level, total antioxidant capacity (T-AOC), and activities of superoxide dismutase (SOD), glutathione peroxidase (GPx), and catalase (CAT) using commercial reagent kits (Beyotime Biotechnology, Shanghai, China). The protein content in the supernatants was measured using a Bicinchoninic Acid (BCA) Protein Assay Kit (Boster Biotechnology, Wuhan, China). All experimental procedures were performed based on the manufacturer’s instructions. The result for oxidative status was normalized to the protein concentration in each sample.

### 2.5. Testicular Morphology Analysis and Immunohistochemistry

For optical microscopy, after fixation in Davidson’s Fixative for 24 h, the testes were dehydrated, paraffin-embedded, and then sectioned at 5 µm with a Leica RM2235 microtome (LEICA, Wetzlar, Germany). The sections were stained with hematoxylin and eosin (HE) and then observed and photographed under an Olympus simon-01 microscope (Olympus Optical Co., Ltd., Beijing, China). For each rooster, both the seminiferous epithelial height and seminiferous tubule diameter were measured ten times from different seminiferous tubules per section. For the spermatogenesis study, the germinal epithelium was scored in the seminiferous tubules according to our previous study [20]. In brief, each tubular cross-section was assigned a grade from 1 to 5 based on the following standard (Table S2).

### 2.6. RNA Sequencing

Three testicular samples were randomly selected from the CON, HPB, and AHP groups, respectively. The RNAiso Plus (Takara Bio Inc., Dalian, China) was used to isolate the total RNA of rooster testes. Library construction and sequencing were primarily conducted by Shanghai Majorbio Bio-pharm Technology Co., Ltd. Transcriptome sequencing was performed using the Illumina NovaSeq 6000 sequencing platform. The Illumina TruSeq™ RNA Sample Prep Kit was utilized for library preparation. Following library construction, quantification was carried out using a PicoGreen dsDNA assay on a TBS-380 fluorometer (Turner Biosystems, Sunnyvale, CA, USA) (Picogreen), and the libraries were diluted accordingly. The insert size was assessed using the Agilent 2100 Bioanalyzer. Subsequently, the library concentration was accurately quantified via fluorescence quantitative PCR to ensure library quality. Libraries were pooled in equimolar ratios and subjected to sequencing on the Illumina platform, generating 150 bp paired-end reads. On the Illumina sequencing platform, the acquired image data were converted into base calls using CASAVA base calling and saved in FASTQ format as raw data. The raw data were demultiplexed based on index sequences to distinguish data from individual samples. Raw reads containing adapter sequences, reads with ambiguous bases (N), and low-quality reads were filtered out. Quality metrics, including Q20, Q30, and GC contents, were calculated for the clean data.

### 2.7. Bioinformatic Analysis

The raw data of FASTQ reads were processed through in-house Perl scripts to obtain clean data. All clear data were mapped to the *Gallus_gallus-5.0* (Ensembl release 94) using Hisat2. The DESeq2 R package (1.16.1) was used to analyze the differentially expressed genes (DEGs) under the following criteria: |fold change| > 2 and False Discovery Rate (FDR) ≤ 0.05. Function and pathway analyses were performed using Kyoto Encyclopedia of Genes and Genomes (KEGG) annotation for the DEGs using the cluster Profiler R package.

### 2.8. Quantitative Real-Time PCR (qRT-PCR)

After extraction, the RNA of each sample was reversed-transcribed to generate cDNA using a HiScript II 1st Strand cDNA Synthesis Kit with a gDNA wiper (Vazyme Biotech, Nanjing, China). The primer sequences of the selected genes were synthesized by Sangon Biotech (Sangon Biotech Co., Ltd., Shanghai, China) and are shown in Table 1. The qRT-PCR assays were carried out using the ChamQ TM SYBR qPCR Master Mix (Vazyme Biotech, Nanjing, China) on the QuantStudio 5 Real-Time PCR System (Thermo Scientific, Sunnyvale, CA, USA). Each gene was repeated three times, and the relative mRNA expression levels were analyzed using the 2^−∆∆Ct^ method, with *GAPDH* as a housekeeping gene.

### 2.9. Statistical Analysis

Data were statistically analyzed using SPSS (version 24.0) and are expressed as the mean ± SEM. Data normality was confirmed using the Shapiro–Wilk test, and the homogeneity of variance was verified through the Levene variance homogeneity test. When variables had groups with normality and homogeneity of variances, a one-way analysis of variance (ANOVA) was used to test the statistical differences among the various experimental groups. Subsequently, Tukey’s post hoc tests were conducted for multiple comparisons among the groups. The ANOVA with Welch’s correction was used in no-normality and no-homogeneity of variances. The statistical differences were considered significant at *p* < 0.05.

## 3. Results

### 3.1. Effect of ALA on Testicular Weights and Sperm Quality in Pb-Exposed Roosters

At the end of the experiment, the roosters in each group were in good overall condition, with no mortality observed. Both end body weight and body weight gain did not show significant differences among the groups. Similarly, there were no significant differences in testis weight and testis index between the six groups (Table 2). As shown in Table 3, six parameters were measured to evaluate the effect of ALA supplementation on mitigating Pb-induced poor semen quality. Sperm concentration and membrane functional integrity were significantly decreased in roosters exposed to Pb compared to the CON group (*p* < 0.05). ALA supplementation mildly improved the alterations in sperm concentration and membrane functional integrity induced by Pb. Furthermore, the HPB group exhibited a higher percentage of abnormal sperm and lower sperm viability than the CON group (*p* < 0.05). ALA significantly ameliorated the decrease in sperm viability in the HPB group but did not significantly affect the percentage of sperm abnormality.

### 3.2. Effect of ALA on Serum Testosterone Levels and Testicular Pb Content in Pb-Exposed Roosters

**Figure 1:**
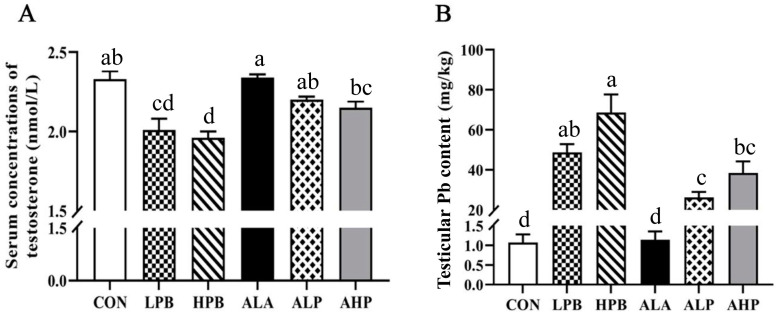
The effects of ALA supplementation on serum testosterone levels and the testicular Pb content in Pb-exposed roosters. (**A**) A statistical analysis of serum testosterone levels. (**B**) A statistical analysis of the testicular Pb content. CON, roosters fed a basal diet; LPB, roosters fed a basal diet supplemented with 150 mg/kg (CH_3_OO)_2_Pb; LPB, roosters fed a basal diet supplemented with 300 mg/kg (CH_3_OO)_2_Pb; ALA, roosters fed a basal diet supplemented with 300 mg/kg ALA; ALP, roosters fed a basal diet supplemented with 300 mg/kg ALA and 150 mg/kg (CH_3_OO)_2_Pb; AHP, roosters fed a basal diet supplemented with 300 mg/kg ALA and 300 mg/kg (CH_3_OO)_2_Pb. The data are presented as means ± SEM (n = 8). Bars with different letters indicate significant differences (*p* < 0.05).

Serum testosterone from roosters was measured using the ELISA method, and the results show stable repeatability and high consistency. The intra-assay coefficient of variation (CV) and the inter-assay CV were <10%. Compared with the LPB group, serum testosterone was obviously increased in roosters in the ALP group (*p* < 0.05). Compared with the HPB group, serum testosterone was significantly increased in roosters in the AHP group (*p* < 0.05). Therefore, ALA treatment can alleviate the Pb-induced decrease in serum testosterone in breeding roosters (Figure 1A).

The Pb levels in the testes of roosters were detected using ICP-MS technology. As shown in Figure 1B, testicular Pb levels were increased in a dose-dependent manner, and the Pb-exposed groups showed a significant increase as compared with the CON and ALA groups (*p* < 0.05). The testicular Pb level of the HPB group was higher than that of the LPB group, but there was no significant difference between the HPB and LPB groups. The ALP group showed significantly lower Pb content compared to the LPB group (*p* < 0.05).

### 3.3. Effect of ALA on Testicular Morphology in Pb-Exposed Roosters

**Figure 2:**
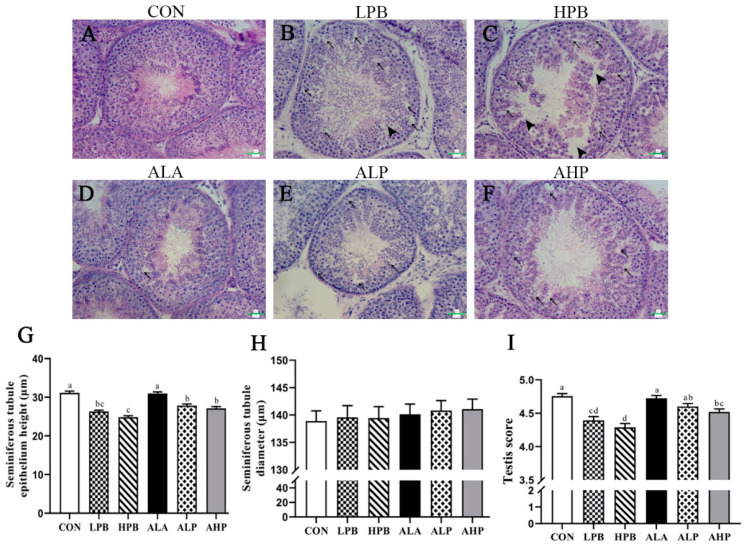
The effects of ALA supplementation on testicular histomorphology in Pb-exposed roosters. (**A**–**F**) HE images of the testis. (**G**) A statistical analysis of the seminiferous epithelium height. (**H**) A statistical analysis of the seminiferous tubule diameter. (**I**) A statistical analysis of the testis scores. CON, basal diet; LPB, basal diet supplemented with 150 mg/kg (CH_3_OO)_2_Pb; LPB, basal diet supplemented with 300 mg/kg (CH_3_OO)_2_Pb; ALA, basal diet supplemented with 300 mg/kg ALA; ALP, basal diet supplemented with 300 mg/kg ALA and 150 mg/kg (CH_3_OO)_2_Pb; AHP, basal diet supplemented with 300 mg/kg ALA and 300 mg/kg (CH_3_OO)_2_Pb. The triangle indicates spermatogenic cell shedding, and the arrow indicates vacuolation of the spermatogenic epithelium. Scale bar = 20 μm. The data are presented as means ± SEM (n = 8). Bars with different letters denote significant differences (*p* < 0.05).

HE staining revealed disorganized vacuolization of the seminiferous epithelium, thinning and rupturing of the seminiferous cell layer, and a decrease in the number of spermatogenic cells in Pb-exposed roosters (Figure 2B,C). Additionally, both the height and testis score of the seminiferous epithelium were significantly decreased in the Pb-exposed groups (Figure 2I). However, Pb did not affect the seminiferous tubule diameter (Figure 2H). Interestingly, ALA intervention significantly improved Pb-induced spermatogenesis disorders, leading to marked increases in the testis score (Figure 2I) and the height of the seminiferous epithelium (Figure 2G).

### 3.4. Effect of ALA on Testicular Oxidative Status in Pb-Exposed Roosters

**Figure 3:**
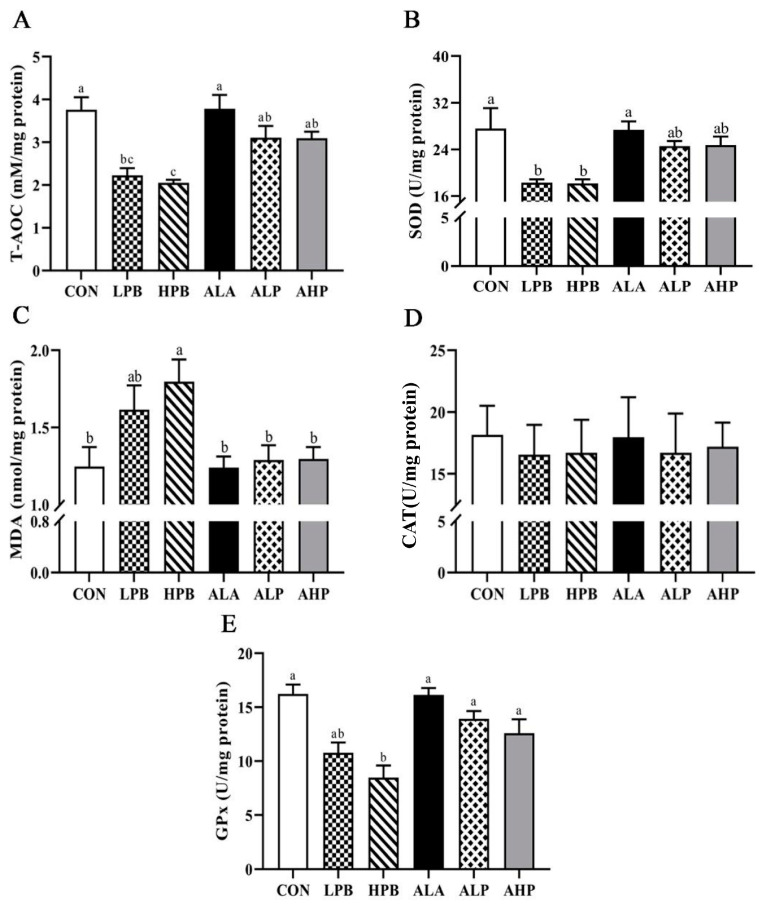
Effects of ALA supplementation on testicular oxidative status in Pb-exposed roosters. (**A**) Total antioxidant capacity of the testis. (**B**) Superoxide dismutase activity of the testis. (**C**) Testicular malondialdehyde content. (**D**) Testicular catalase activity. (**E**) Glutathione peroxidase activity of the testis. T-AOC, total antioxidant capacity; SOD, superoxide dismutase; MDA, malondialdehyde; CAT, catalase; GPx, glutathione peroxidase. Groups: CON, basal diet; LPB, basal diet supplemented with 150 mg/kg (CH_3_OO)_2_Pb; LPB, basal diet supplemented with 300 mg/kg (CH_3_OO)_2_Pb; ALA, basal diet supplemented with 300 mg/kg ALA; ALP, basal diet supplemented with 300 mg/kg ALA and 150 mg/kg (CH_3_OO)_2_Pb; AHP, basal diet supplemented with 300 mg/kg ALA and 300 mg/kg (CH_3_OO)_2_Pb. Data are presented as means ± SEM (n = 8). Bars with different letters indicate significant differences (*p* < 0.05).

Figure 3 shows the effect between ALA supplement and Pb challenge in the concentrations of testis oxidation and antioxidant indicators. Compared with the CON group, Pb exposure remarkably decreased the T-AOC levels and activities of SOD and GPx (Figure 3). Moreover, MDA was markedly increased in the HPB group (*p* < 0.05) but not significantly elevated in the LPB group compared to the CON and ALA-treated groups. Roosters receiving ALA ameliorated the stimulation of Pb on antioxidant status. The AHP group exhibited a lower MDA content but a higher T-AOC level, and the activities of GPx and SOD were higher than those in the HPB group (*p* < 0.05).

### 3.5. Effect of ALA on DEGs Expression in Testes in Pb-Exposed Roosters

In order to further investigate the potential key molecular mechanisms underlying the ALA alleviation of Pb-induced testicular damage, we selected testes samples from the CON, HPB, and AHP groups for RNA-seq. As shown in Table 4, nine libraries were constructed for RNA sequencing, with averages of 52,690,622, 60,097,317.3, and 61,477,925.3 clean reads obtained from the CON, HPB, and APB groups, respectively. The base Q20 exceeded 98.18%, and the base Q30 exceeded 94.78%, indicating that the sequencing data from the nine libraries met the necessary quality standards for subsequent bioinformatics analysis. The total rate of mapped clean reads ranged from 91.46% to 92.17%, while the proportion of uniquely mapped reads ranged from 88.46% to 89.15%.

**Figure 4:**
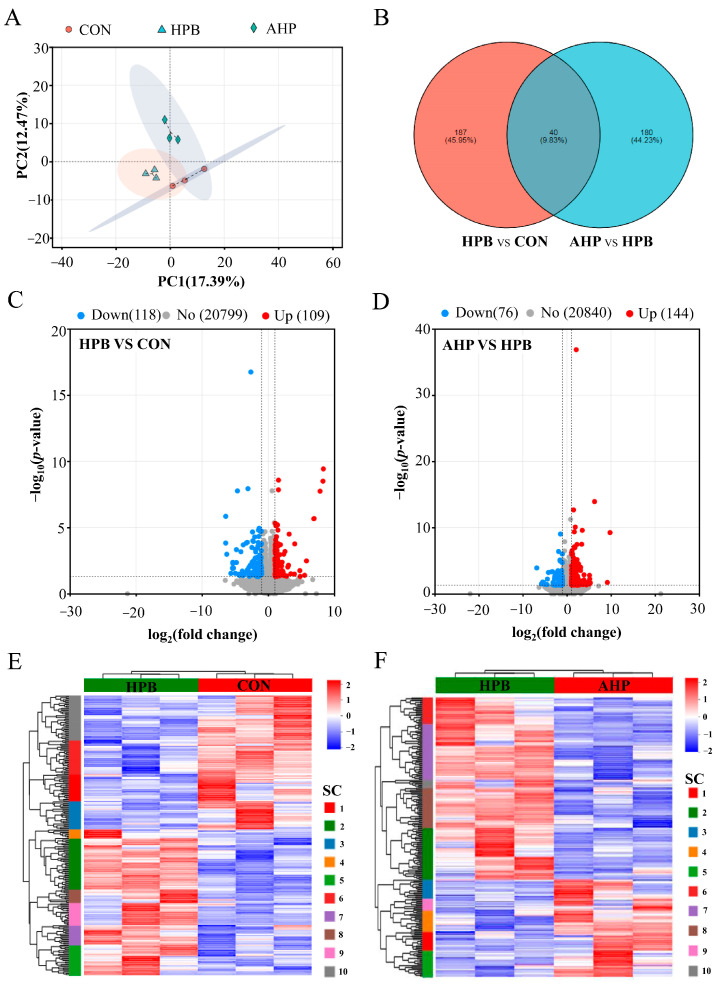
Effects of ALA supplementation on DEG expression in Pb-exposed roosters. (**A**) PCA plot of sample distribution. (**B**) Venn diagram of DEGs. (**C**,**D**) Volcano plot of significant DEGs detected from data sets. (**E**,**F**) Heatmaps showing distribution of DEGs in data sets. Groups: CON, basal diet; HPB, basal diet supplemented with 300 mg/kg (CH_3_OO)_2_Pb; AHP, basal diet supplemented with 300 mg/kg ALA and 300 mg/kg (CH_3_OO)_2_Pb. SC, subcluster.

A principal component analysis (PCA) revealed that samples between groups were dispersed, while samples within groups were clustered (Figure 4A). The volcano plot identified 227 differentially expressed genes (DEGs), including 109 up-regulated genes and 118 down-regulated genes in the HPB group compared to the CON group (Figure 4C). Similarly, 220 DEGs, including 144 up-regulated and 76 down-regulated genes, were identified in the APB group as compared to the HPB group (Figure 4D). The Venn diagram shows that 40 common DEGs were shared between the HPB vs. CON and APB vs. HPB comparisons (Figure 4B). As shown in Figure 4E and Figure 4F, DEGs were hierarchically clustered into ten distinct subclusters, clearly demonstrating a significant difference in the testicular expression profiles between the HPB and CON groups, as well as between the APB and HPB groups.

### 3.6. KEGG Enrichment Analysis of DEGs Between Groups of Rooster Testes

**Figure 5:**
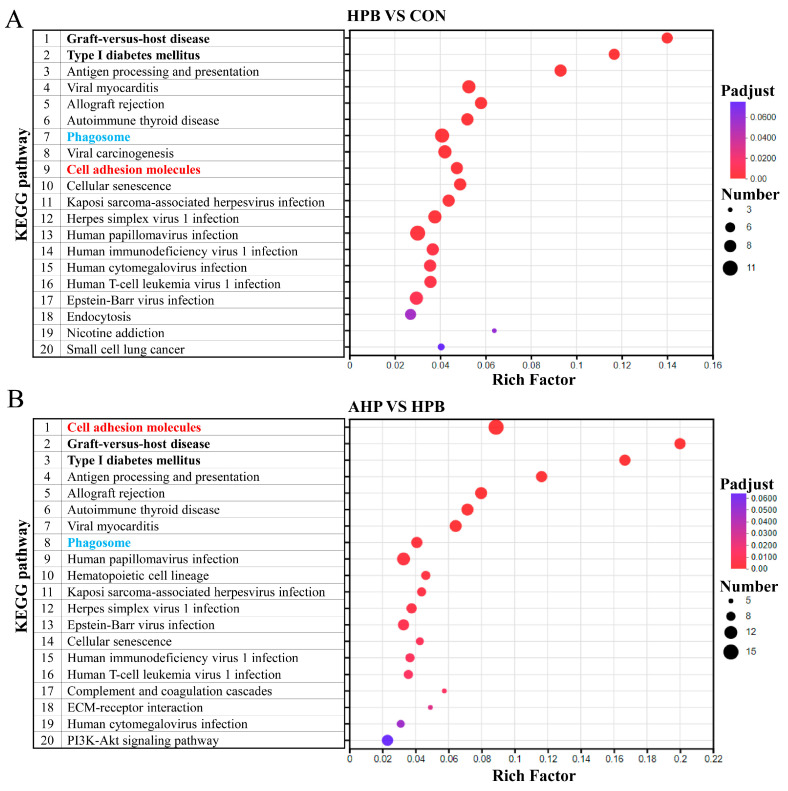
A KEGG pathway enrichment analysis of DEGs in two comparison groups of rooster testes. (**A**) HPB vs. CON. (**B**) AHP vs. HPB. Groups: CON, basal diet; HPB, basal diet supplemented with 300 mg/kg (CH_3_OO)_2_Pb; AHP, basal diet supplemented with 300 mg/kg ALA and 300 mg/kg (CH_3_OO)_2_Pb. The X-axis represents the rich factor, which is the ratio of the number of genes enriched in this pathway to the number of annotated genes. A larger rich factor indicates a higher degree of enrichment. The Y-axis represents the pathway. Number: The size of the dots represents the number of genes in this pathway. Padjust: the *p*-value corrected using the Benjamini–Hochberg method. Padjust < 0.05 was considered to be statistically significant. The colors of the dots correspond to different Padjust ranges. The top 20 enrichment results are displayed under the premise that Padjust < 1.

A KEGG enrichment analysis was performed to pinpoint key genes and pathways involved in the biological functions and molecular interactions of the DEGs. The top 20 KEGG pathways from the pairwise comparison are shown in Figure 5, respectively. Eighteen key pathways were significant in the comparison between CON and HPB, while nineteen key pathways were significant in the comparison between HPB and APH (Padjust < 0.05). In the comparison between CON and HPB, key characteristic genes, such as *MARCO* and *ITGA2*, were identified within the phagosome pathway. The *CD8A* gene was identified in the pathway of cell adhesion molecules, while the *CDKN2A* gene was revealed in the cellular senescence pathway. The related pathways of virus infection disease, such as herpes simplex virus 1 infection, human papillomavirus infection, and Epstein–Barr virus infection, were enriched, with *TBK1* identified as a pivotal gene. Similarly, the pathways of cell adhesion molecules, phagosome, cell senescence, immune disease, and virus infection disease were also enriched in the comparison between HPB and AHP. Key characteristic genes, such as *CLDN19* (*claudin 19*), *CD86*, *CNTN2*, and *ITGA4*, were identified in the pathway of cell adhesion molecules. The *RASSF5* gene was identified in the cellular senescence pathway, while the *MARCO* and *ITGA2* genes were revealed in the phagosome pathway. In addition, the related pathways of virus infection disease, such as kaposi sarcoma-associated herpesvirus infection, human papillomavirus infection, and Epstein–Barr virus infection, were enriched, with *FAS* and *TBK1* being identified as pivotal genes. The characteristic genes filtered are shown in the Supplementary Files (Table S3).

### 3.7. Detection of DEGs by qRT-PCR

**Figure 6:**
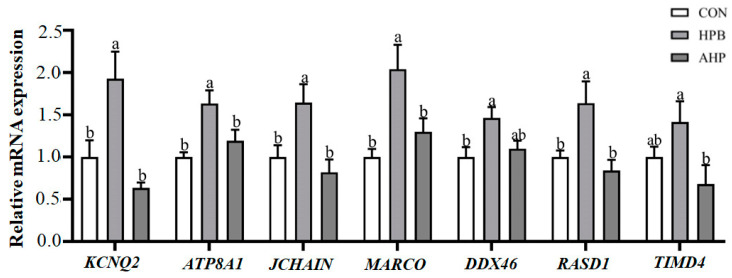
The relative mRNA expression of DEGs in rooster testes. Groups: CON, roosters fed a basal diet; HPB, roosters fed a basal diet supplemented with 300 mg/kg (CH_3_OO)_2_Pb; AHP, roosters fed a basal diet supplemented with 300 mg/kg ALA and 300 mg/kg (CH_3_OO)_2_Pb. The data are presented as means ± SEM (n = 3). Bars with different letters indicate significant differences (*p* < 0.05).

As shown in Figure 6, seven DEGs were selected and used to determine the reliability of RNA-seq. Those genes play vital roles in spermatogenesis and immune function in the testes. The *KCNQ2*, *ATP8A1*, *JCHAIN*, *MARCO*, *DDX46*, and *RASD1* genes increased in the HPB group as compared with the CON group (*p* < 0.05). Dietary ALA decreased the relative mRNA expression of *KCNQ2*, *ATP8A1*, *JCHAIN*, *MARCO*, and *TIMD4* (*p* < 0.05). The results of the qRT-PCR show that the data from the qRT-PCR are in accordance with the results of transcriptome sequencing, and they further indicate that RNA-seq was reliable in the testis transcriptome (Figure S1).

## 4. Discussion

Lead is a toxic heavy metal that contaminates the environment, as well as feed and food, and is recognized as a major male reproductive toxicant. This study demonstrates that Pb exposure is associated with an increased risk of male infertility. Dietary Pb supplementation can accumulate in the testes, causing testicular damage and impairing spermatogenesis. In rooster testes, Pb exposure induced testicular dysfunction by triggering oxidative stress and autophagy [14]. Natural antioxidants have been shown to mitigate the toxicity of heavy metals [21,22], and a previous study reported that ALA interfered with Cd^2+^ accumulation in the testes and restored antioxidant balance, thereby preventing Cd^2+^-induced testicular damage [13]. In the present study, Pb exposure led to testicular Pb accumulation and significant reductions in serum testosterone and semen quality, along with damage to the germinal epithelium, indicating that Pb exposure caused testicular toxicity. A diet supplemented with ALA was found to mitigate Pb-induced reproductive damage in roosters, suggesting its potential as a therapeutic agent for alleviating Pb-induced testicular dysfunction.

Ninety days of Pb exposure (200 mg/L Pb^2+^ in water, approximately equal to the values of 25 mg/kg BW/day) did not change the water consumption, feed intake, body weight, and testicular organ index of mice compared with the group without Pb exposure [23]. In this study, there were no significant differences in body weight, testicular weight, or the testicular organ index among the rooster groups, indicating that overall, the metabolism of Pb-exposed roosters was in the normal range. However, rats that were orally administered (CH_3_OO)_2_Pb (100 or 500 mg/kg BW/day) showed significant decreases in body weight and testicular weight [24,25]. The explanation for the different results was that the roosters in this study were exposed to a low dose of (CH_3_OO)_2_Pb (150–300 mg/kg in diet, approximately equal to the values of 5–10 mg/kg BW/day), which might have made them less susceptible to Pb exposure. In the present study, the testicular Pb content increased in a dose-dependent manner, and the Pb-treated groups exhibited significantly higher Pb levels in the testes than the CON group, indicating that eight weeks of Pb exposure resulted in Pb accumulation in rooster testes. No significant difference was tested in the testicular Pb content between the HPB and LPB groups, suggesting that testicular cells might have a limited capacity to accommodate Pb^2+^ and that a further increase in Pb^2+^ within testicular cells possibly causes cell death and reduces spermatogenic cells [23]. It has been shown that ALA, as a metal chelator, can bind to Pb^2+^, Fe^2+^, and Cu^2+^ ions [26,27]. Additionally, dietary ALA could reduce the accumulation levels of Pb in the liver, kidney, and muscle of *Channa argus* [28]. In this study, testicular Pb levels decreased in the AHP group compared to the HPB group, which might have resulted from the use of ALA, which appeared to counteract this accumulation by chelating Pb.

**Figure 7:**
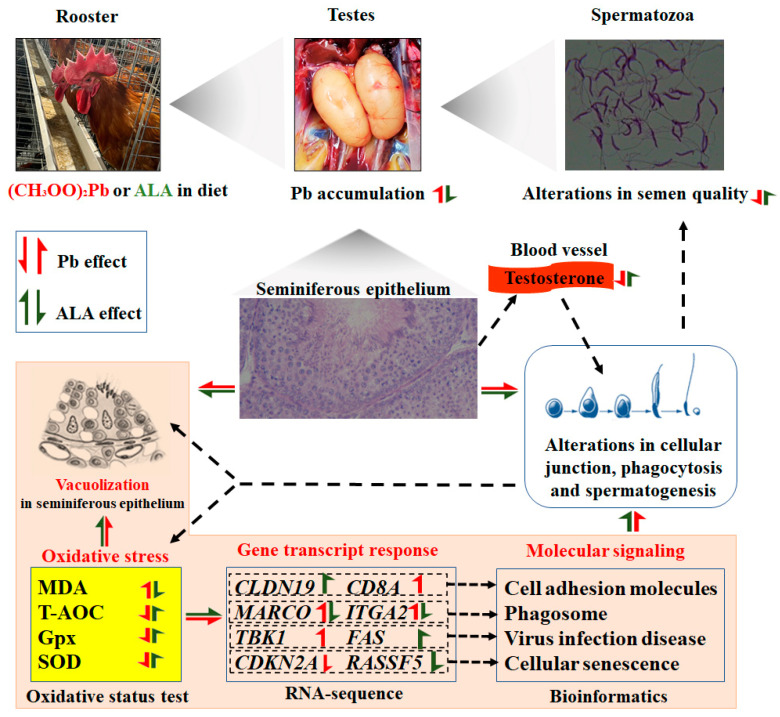
Hypothetical molecular model of how ALA alleviates Pb-induced testicular damage and semen quality by reducing oxidative stress and regulating key signal pathways. Dotted arrows represent promotion.

Pb exposure was associated with a disruption in the testicular structure and increased abnormal sperm in rat and mice [29,30]. Similarly, in our study, Pb exposure reduced the height of the spermatogenic epithelium, increased the percentage of abnormal sperm, and induced the decrease in serum testosterone levels, sperm viability, sperm concentration, and membrane functional integrity of rooster. These results suggest that Pb interfered with spermatogenesis and caused poor semen quality in the roosters. Recent studies found that exposure to heavy metals (Hg, Ni, Cu, and Cd) can induce significant alterations in the properties of protamine-like protein within mussel spermatozoa, which help to compact DNA during spermatogenesis, thereby making sperm DNA more condensed and facilitating sperm motility and morphology [31,32]. In the present study, Pb exposure might cause alterations in many structural or functional components of rooster spermatozoa, such as protamine-like protein. These alterations possibly further led to changes in the sperm motility, abnormality, and membrane integrity of rooster sperm. However, this needs to be verified by further experiments (Figure 7). Our previous studies have demonstrated that dietary ALA supplementation enhanced serum testosterone levels, improved sperm quality, and promoted testicular spermatogenesis in aged breeder roosters [12]. In the present study, dietary ALA intervention alleviated the reduced vacuolization in testicular tissue and the decrease in testosterone levels and sperm quality, indicating that dietary ALA effectively ameliorated Pb-induced testicular dysfunction in roosters.

The blood–testis barrier (BTB) provides a unique microenvironment to maintain testicular material exchange and spermatogenesis, and testicular function is dependent on the microenvironment and biological barrier established by the BTB [33]. A recent study reported that Pb exposure can disrupt testicular BTB integrity by modifying the expression of tight junction-related genes and reducing the occluding protein in mice [15]. Oxidative stress was considered as a critical mechanism of BTB injury, and excessive accumulation of ROS in the testis destroyed the integrity of the BTB [34]. One of the main mechanisms of Pb toxicity is oxidative stress, where Pb induces excessive cellular production of ROS, which imbalances the antioxidant system and leads to testicular damage [25]. In rooster testes, Pb exposure increased the testicular MDA content; decreased the activities of SOD, glutathione-S-transferase, and GPx; and evoked oxidative stress [14]. In the present study, Pb exposure caused an excessive accumulation of MDA and a decrease in T-AOC levels, and it reduced the activities of SOD and GPx enzymes in the testes. These findings suggest that Pb exposure disrupted the redox environment of the rooster testes and induced testicular oxidative stress, which further damaged cellular junction and BTB integrity (Figure 7). ALA, known for its strong antioxidant properties, has been shown to enhance antioxidant defenses in rooster testes [12]. In this study, ALA supplementation reduced the MDA content and increased the activities of GPx and SOD enzymes in the testes compared to the Pb-exposed groups. These results demonstrate that ALA effectively eliminated the overproduction of MDA, restored antioxidant enzyme activity, maintained T-AOC levels, and alleviated Pb-induced oxidative stress.

The RNA-seq results reveal that DEGs in both the HPB vs. CON and AHP vs. HPB comparisons were enriched in the cell adhesion molecules pathway. In testicular tissue, cell adhesion occurred through cell junctions, with cell adhesion molecules playing a crucial role in this process [35]. Up-regulating cell junction-related genes and proteins can preserve the BTB’s ultra-structure and mitigate germinal epithelium compartment alterations [36]. In this study, the genes associated with tight junctions (*CLDN 19*) in the AHP group were significantly up-regulated compared to those in the HPB group, further confirming the alleviating effect of ALA on Pb-induced BTB damage. Another enriched pathway was the phagosome, which is a cellular process involving the ingestion and elimination of particles larger than 0.5 μm in diameter, including microorganisms, foreign substances, and apoptotic cells [37]. *MARCO*, expressed in testicular macrophages, maintains testicular immune homeostasis by clearing pathogens, damaged cells, and cell debris that could impede spermatogenesis, thereby fostering a stable microenvironment for sperm development and maturation [38]. In this study, the relative mRNA expression of the *MARCO* gene was significantly increased in the HPB group and significantly decreased in the AHP group. Meanwhile, the RNA-seq results also confirm this expression trend of the *MARCO* gene. These findings suggest that Pb exposure activated the phagosome pathway in the testes to promptly clear Pb-induced tissue waste via phagocytosis (Figure 7). Dietary ALA down-regulated the relative mRNA expression of the *MARCO* gene and alleviated the activation of the phagosome pathway caused by Pb exposure, demonstrating a protective effect.

ROS are produced during normal metabolism and play vital roles in many cellular processes, such as ion transport, immunomodulation, cytokine transcription, and apoptosis [39]. In general, there is a balance between ROS production and its detoxification by an enzymatic antioxidant system consisting of SOD, GPx, and CAT [40]. During viral infections, the production of ROS was increased and exerted antimicrobial action to kill viral infections via autophagy and apoptosis [41]. Meanwhile, viruses prevented the synthesis of SOD, GPx, and CAT, leading to redox imbalance and cellular oxidative stress, thereby activating the immune response and causing chronic diseases such as diabetes mellitus and autoimmune diseases [42]. In this study, a bioinformatics analysis enriched several signal pathways of virus infection and disease, including virus infection pathways, autoimmune thyroid disease, graft-versus-host disease, and type I diabetes mellitus, suggesting that chronic Pb exposure induced oxidative stress and might have further activated the related pathways of virus infection and disease, diabetes mellitus, and autoimmune disease. As an antioxidant, ALA supplementation may be a promising factor to attenuate the activation of these molecular pathways using Pb-induced oxidative stress in rooster (Figure 7). Oxidative stress can also induce cellular senescence, while antioxidant treatment can delay senescence process [43]. In this study, Pb exposure down-regulated the gene expression of *CDKN2A*, suggesting that chronic Pb exposure did not induce testicular cell senescence. Dietary ALA supplementation decreased the gene expression of *RASSF5*, indicating that ALA might enhance cell proliferation in rooster testes. However, no obvious difference was found in testes weight and testis index, implying that both Pb exposure and ALA supplementation had a limited impact on the proliferation and senescence of testicular cells.

The results of the validation of DEGs show that Pb exposure significantly up-regulated the gene expression of *KCNQ2*, *DDX46*, and *ATP8A1*. These three genes primarily modulated cellular ion homeostasis [44], cell proliferation [45], and the structural stability of the sperm membrane [46]. With Pb exposure, the subsequent up-regulation of these genes possibly influenced sperm structure, motility, and viability, leading to reduced sperm motility and membrane integrity as well as elevated sperm abnormality. In the AHP group, the expression levels of these genes showed no significant differences compared with those in the CON group, indicating that the addition of ALA alleviated the adverse effects of Pb exposure and stabilized the expression of these three genes. *RASD1* can influence the proliferation of testicular Sertoli cells through its response to the plasticizer and the down-regulation of *RASD1* expression can facilitate the proliferation of these cells [47]. In this study, Pb exposure induced an increase in the gene expression of *RASD1*, while ALA attenuated the up-regulation of *RASD1* expression. These results suggest that ALA might alleviate the stimulation of Pb on testicular tissue and help to stabilize the proliferative state of testicular tissue cells.

## 5. Conclusions

This study demonstrated that ALA supplementation alleviated Pb-induced testicular toxicity in roosters. Pb exposure led to reduced serum testosterone levels, impaired semen quality, and damage to the seminiferous epithelium, while ALA supplementation improved serum testosterone levels, sperm concentration, membrane integrity, and reduced testicular Pb accumulation. A histological analysis showed an enhanced seminiferous epithelium height and reduced oxidative stress. A KEGG analysis identified key pathways, including the phagosome and cell adhesion molecules pathways, as well as pathways related to immune function and viral infection disease. Based on DEGs, characteristic genes such as *KCNQ2*, *ATP8A1*, *JCHAIN*, *MARCO*, *DDX46*, and *RASD1* were up-regulated by Pb exposure but were attenuated by ALA treatment. Overall, ALA effectively mitigated Pb-induced testicular damage in rooster, suggesting its potential as a therapeutic agent for Pb toxicity in birds and other species.

## Figures and Tables

**Table 1:** Primer sequences used for RT-qPCR.

Gene	Primer Sequence (5′→3′)	Amplicon Size (bp)	Accession Number
*KCNQ2*	forward: GGCAGAACTCCGAAGAAGCA reverse: CTGACTTTCAGTCCTGGCGT	109	XM_015296640.4
*ATP8A1*	forward: TGCTGACACTGTACTGCTCT reverse: ATGTTAGGGGCAAACCCTGTC	113	XM_025149963.3
*JCHAIN*	forward: TTCGTCCTTGTGGCAGGTTATC reverse: TGTCTTTGGAGGGGACGAAC	109	NM_205064.1
*MARCO*	forward: TGCTGGCGTACAAAGTGTTT reverse: TCTCTTCGGCATGGAAAGCA	78	NM_204736.3
*DDX46*	forward: CGGGAGTCCAGGCACTATC reverse: GCGTCTGTCCTCACGTTTTC	105	NM_001389392.2
*RASD1*	forward: GAGGACTTCCACCGCAAGTT reverse: GAGGATGAAAACGTCACCTGT	132	NM_001044636.2
*TIMD4*	forward: GGAACAGGGTGACGTTCAGA reverse: CATCAATGGAGGTGCTTCGAG	197	NM_001006149.2
*GAPDH*	forward: TGATGCCCCCATGTTTGTGA reverse: TGGCATGGACAGTGGTCATA	164	NM_204305.1

Note: *KCNQ2*, potassium voltage-gated channel subfamily Q member 2; *ATP8A1*, ATPase phospholipid transporting 8A1; *JCHAIN*, joining chain of multimeric IgA and IgM; *MARCO*, macrophage receptor with collagenous structure; *DDX46*, DEAD-box helicase 46; *RASD1*, ras-related dexamethasone-induced 1; *TIMD4*, T cell immunoglobulin and mucin domain containing 4.

**Table 2:** Effects of ALA supplementation on body weight and testicular organ index of Pb-exposed roosters.

Items	Treatments					
	CON	LPB	HPB	ALA	ALP	AHP
Initial body weight (kg)	2.83 ± 0.03	2.81 ± 0.05	2.81 ± 0.08	2.80 ± 0.05	2.80 ± 0.09	2.81 ± 0.06
End body weight (kg)	3.38 ± 0.06	3.33 ± 0.08	3.26 ± 0.12	3.31 ± 0.08	3.26 ± 0.16	3.34 ± 0.09
Body weight gain (kg)	0.55 ± 0.04	0.52 ± 0.04	0.45 ± 0.06	0.51 ± 0.04	0.47 ± 0.08	0.53 ± 0.03
Testis weight (g)	39.70 ± 2.94	38.12 ± 2.84	35.36 ± 1.73	38.05 ± 2.10	37.87 ± 2.72	39.46 ± 4.49
Testis index (%)	1.17 ± 0.08	1.16 ± 0.10	1.10 ± 0.08	1.16 ± 0.08	1.15 ± 0.04	1.18 ± 0.14

Note: CON, roosters fed a basal diet; LPB, roosters fed a basal diet supplemented with 150 mg/kg (CH_3_OO)_2_Pb; LPB, roosters fed a basal diet supplemented with 300 mg/kg (CH_3_OO)_2_Pb; ALA, roosters fed a basal diet supplemented with 300 mg/kg ALA; ALP, roosters fed a basal diet supplemented with 300 mg/kg ALA and 150 mg/kg (CH_3_OO)_2_Pb; AHP, roosters fed a basal diet supplemented with 300 mg/kg ALA and 300 mg/kg (CH_3_OO)_2_Pb. The values are shown as means ± SEM (n = 8).

**Table 3:** Effects of ALA supplementation on semen quality of Pb-exposed roosters.

Items	Treatments					
	CON	LPB	HPB	ALA	ALP	AHP
Ejaculate volume (mL)	0.84 ± 0.07	0.59 ± 0.06	0.70 ± 0.05	0.85 ± 0.07	0.78 ± 0.09	0.75 ± 0.07
Sperm concentration (10^9^ cells/mL)	3.18 ± 0.20 ^a^	2.14 ± 0.17 ^bc^	2.04 ± 0.20 ^c^	3.11 ± 0.22 ^a^	2.84 ± 0.11 ^ab^	2.81 ± 0.14 ^ab^
Abnormal sperm (%)	5.25 ± 0.21 ^a^	5.75 ± 0.25 ^ab^	6.25 ± 0.25 ^b^	5.31 ± 0.23 ^ab^	5.50 ± 0.19 ^ab^	5.88 ± 0.25 ^ab^
Sperm viability (%)	84.06 ± 0.46 ^ab^	81.88 ± 0.53 ^bc^	80.78 ± 0.82 ^c^	84.53 ± 0.62 ^a^	83.44 ± 0.57 ^ab^	82.99 ± 0.40 ^ab^
pH	7.58 ± 0.41	7.51 ± 0.48	7.50 ± 0.42	7.58 ± 0.42	7.59 ± 0.48	7.49 ± 0.40
Membrane functional integrity (%)	94.81 ± 0.17 ^a^	93.68 ± 0.38 ^bc^	93.37 ± 0.30 ^c^	94.77 ± 0.13 ^a^	94.50 ± 0.25 ^ab^	94.41 ± 0.11 ^ab^

Note: CON, roosters fed a basal diet; LPB, roosters fed a basal diet supplemented with 150 mg/kg (CH_3_OO)_2_Pb; LPB, roosters fed a basal diet supplemented with 300 mg/kg (CH_3_OO)_2_Pb; ALA, roosters fed a basal diet supplemented with 300 mg/kg ALA; ALP, roosters fed a basal diet supplemented with 300 mg/kg ALA and 150 mg/kg (CH_3_OO)_2_Pb; AHP, roosters fed a basal diet supplemented with 300 mg/kg ALA and 300 mg/kg (CH_3_OO)_2_Pb. The values are shown as means ± SEM (n = 8). Means with different letters differ significantly (*p* < 0.05).

**Table 4:** RNA-seq read statistics of rooster testes.

Sample	Clean Reads	Clean Bases	Q20	Q30	GC Content	Total Mapped	Uniquely Mapped
CON1	50,015,498	7,372,621,148	98.22%	94.91%	52.22%	92.17%	89.04%
CON2	56,785,098	8,398,140,920	98.18%	94.78%	52.13%	91.45%	88.63%
CON3	51,271,270	7,567,391,561	98.25%	94.90%	51.35%	91.71%	88.96%
HPB1	57,456,806	8,470,844,569	98.24%	94.92%	51.22%	91.8%	89.03%
HPB2	60,906,272	8,983,684,655	98.29%	95.01%	51.69%	91.89%	89.15%
HPB3	61,928,874	9,158,784,735	98.24%	94.89%	51.72%	91.68%	88.81%
AHP1	71,146,770	10,521,995,825	98.27%	94.99%	51.74%	91.57%	88.56%
AHP2	54,704,400	8,120,147,760	98.28%	95.01%	51.66%	91.46%	88.46%
AHP3	58,582,606	8,674,540,804	98.31%	95.09%	52.13%	91.97%	89.14%

Note: Q20, percentage of bases with Phred values higher than 20; Q30, percentage of bases with Phred values higher than 30. Unit of clean bases is bp. Groups: CON, roosters fed basal diet; HPB, roosters fed basal diet supplemented with 300 mg/kg (CH_3_OO)_2_Pb; AHP, roosters fed basal diet supplemented with 300 mg/kg ALA and 300 mg/kg (CH_3_OO)_2_Pb.

## Data Availability

The data supporting the findings of this study are included within the article. Any additional data that support the study’s conclusions can be made available by the corresponding author upon reasonable request.

## Supplementary Materials

The following supporting information can be downloaded at https://www.mdpi.com/article/10.3390/toxics13050341/s1, Figure S1: TPM of selected DEGs in rooster testes; Table S1: Ingredient composition and nutrient levels of basal diet; Table S2: Criteria of spermatogenesis scoring method; Table S3: DEGs identified in KEGG enrichment analysis.

## References

[1] Boskabady M., Marefati N., Farkhondeh T., Shakeri F., Farshbaf A., Boskabady M.H. (2018). The effect of environmental lead exposure on human health and the contribution of inflammatory mechanisms, a review. Environ. Int..

[2] Wirth J.J., Mijal R.S. (2010). Adverse effects of low level heavy metal exposure on male reproductive function. Syst. Biol. Reprod. Med..

[3] Massanyi P., Massanyi M., Madeddu R., Stawarz R., Lukac N. (2020). Effects of cadmium, lead, and mercury on the structure and function of reproductive organs. Toxics.

[4] Godinez-Solis Y., Solis-Heredia M.J., Roa-Espitia A., Parra-Forero L.Y., Hernandez-Gonzalez E.O., Hernandez-Ochoa I., Quintanilla-Vega B. (2019). Low concentrations of lead decrease the sperm fertilization ability by altering the acrosome reaction in mice. Toxicol. Appl. Pharmacol..

[5] Sies H.J.D. (2020). Reactive oxygen species (ROS) as pleiotropic physiological signaling agents. Nat. Rev. Mol. Cell Biol..

[6] Bisht S., Faiq M., Tolahunase M., Dada R. (2017). Oxidative stress and male infertility. Nat. Rev. Urol..

[7] Abbaszadeh S., Yadegari P., Imani A., Taghdir M. (2021). Vitamin D3 protects against lead-induced testicular toxicity by modulating Nrf2 and NF-kappaB genes expression in rat. Reprod. Toxicol..

[8] Al-Megrin W.A., Alomar S., Alkhuriji A.F., Metwally D.M., Mohamed S.K., Kassab R.B., Abdel-Moneim A.E., EI-Khadragy M.F. (2020). Luteolin protects against testicular injury induced by lead acetate by activating the Nrf2/HO-1 pathway. IUBMB Life.

[9] Wang B., Wang Y., Zhang J., Hu C., Jiang J., Li Y., Peng Z. (2023). ROS-induced lipid peroxidation modulates cell death outcome: Mechanisms behind apoptosis, autophagy, and ferroptosis. Arch. Toxicol..

[10] Shanaida M., Lysiuk R., Mykhailenko O., Hudz N., Abdulsalam A., Gontova T., Oleshchuk O., Ivankiv Y., Shanaida V., Lytkin D. (2025). Alpha-lipoic acid: An antioxidant with anti-aging properties for disease therapy. Curr. Med. Chem..

[11] Superti F., Russo R. (2024). Alpha-lipoic acid: Biological mechanisms and health benefits. Antioxidants.

[12] Ye N., Lv Z., Huang Z., Cheng Y., Wei Q., Shi F. (2022). Dietary folic acid supplementation improves semen quality and spermatogenesis through altering autophagy and histone methylation in the testis of aged broiler breeder roosters. Theriogenology.

[13] El-Maraghy S.A., Nassar N.N. (2011). Modulatory effects of lipoic acid and selenium against cadmium-induced biochemical alterations in testicular steroidogenesis. J. Biochem. Mol. Toxicol..

[14] Huang H., Wang M., Hou L., Lin X., Pan S., Zheng P., Zhao Q. (2021). A potential mechanism associated with lead-induced spermatogonia and Leydig cell toxicity and mitigative effect of selenium in chicken. Ecotox. Environ. Saf..

[15] Dolati P., Khodabandeh Z., Zamiri M.J., Jamhiri I., Mehrabani D. (2020). The effect of lead acetate and quercetin on the tight and gap junctions in the mouse testis. Biol. Trace Elem. Res..

[16] (2005). National Criterion of China. Hygienical Standard for Feeds.

[17] Jin X., Liu C.P., Teng X.H., Fu J. (2016). Effects of dietary selenium against lead toxicity are related to the ion profile in chicken muscle. Biol. Trace Elem. Res..

[18] Jin X., Xu Z., Zhao X., Chen M., Xu S. (2017). The antagonistic effect of selenium on lead-induced apoptosis via mitochondrial dynamics pathway in the chicken kidney. Chemosphere.

[19] Zhang J., Wang S., Hao X., Gang S., Xu S. (2020). The antagonistic effect of selenium on lead-induced necroptosis via MAPK/NF-kappaB pathway and HSPs activation in the chicken spleen. Ecotoxicol. Environ. Saf..

[20] Wu H., Ye N., Huang Z., Lei K., Shi F., Wei Q. (2023). Dietary curcumin supplementation relieves hydrogen peroxide-induced testicular injury by antioxidant and anti-apoptotic effects in roosters. Theriogenology.

[21] Vafaee F., Derakhshani M., Rahbardar M.G., Hosseinzadeh H. (2024). Alpha-lipoic acid, as an effective agent against toxic elements: A review. Naunyn-Schmiedeberg’s Arch. Pharmacol..

[22] Long C., Zhao Z.X., Willing B.P., Sheng X.H., Wang X.G., Xiao L.F., Qi X.L. (2024). Alpha-linolenic acid supplementation improves testosterone production in an aged breeder rooster model: Role of mitochondrial modulation and SIRT1 activation. Mol. Nutr. Food Res..

[23] Zhang Z.Y., Yu J., Xie J., Liu D.Y., Fan Y.S., Ma H.T., Wang C., Hong Z. (2021). Improvement roles of zinc supplementation in low dose lead induced testicular damage and glycolytic inhibition in mice. Toxicology.

[24] Kahalerras L., Otmani I., Abdennour C. (2022). Wild garlic allium triquetrum L. alleviates lead acetate-induced testicular injuries in rats. Biol. Trace Elem. Res..

[25] Ahmed H.A., Ali H.A., Mutar T.F. (2021). Protective effects of olive leaf extract against reproductive toxicity of the lead acetate in rats. Environ. Sci. Pollut. Res. Int..

[26] Biewenga G.P., Haenen G.R.M.M., Bast A. (1997). The pharmacology of the antioxidant lipoic acid. Gen. Pharmacol..

[27] Tripathi A.K., Ray A.K., Mishra S.K., Bishen S.M., Mishra H., Khurana A. (2023). Molecular and therapeutic insights of alpha-lipoic acid as a potential molecule for disease prevention. Rev. Bras. Farmacogn..

[28] Li M., Kong Y., Wu X., Yin Z., Niu X., Wang G. (2021). Dietary α-lipoic acid can alleviate the bioaccumulation, oxidative stress, cell apoptosis, and inflammation induced by lead (Pb) in *Channa argus*. Fish Shellfish Immunol..

[29] El-fakharany Y.M., Mohamed E.M., Etewa R.L., Hamid O.I.A. (2022). Selenium nanoparticles alleviate lead acetate-induced toxicological and morphological changes in rat testes through modulation of calmodulin-related genes expression. J. Biochem. Mol. Toxic..

[30] Elsheikh N.A.H., Omer N.A., Yi-Ru W., Mei-Qian K., Ilyas A., Abdurahim Y., Wang G.L. (2020). Protective effect of betaine against lead-induced testicular toxicity in male mice. Andrologia.

[31] Lettieri G., Notariale R., Ambrosino A., Di Bonito A., Giarra A., Trifuoggi M., Manna C., Piscopo M. (2021). Spermatozoa transcriptional response and alterations in PL proteins properties after exposure of *Mytilus galloprovincialis* to mercury. Int. J. Mol. Sci..

[32] Marinaro C., Lettieri G., Chianese T., Bianchi A.R., Zarrelli A., Palatucci D., Scudiero R., Rosati L., De Maio A., Piscopo M. (2024). Exploring the molecular and toxicological mechanism associated with interactions between heavy metals and the reproductive system of *Mytilus galloprovincialis*. Comp. Biochem. Physiol. C Toxicol. Pharmacol..

[33] Su L., Mruk D.D., Cheng C.Y. (2011). Drug transporters, the blood-testis barrier, and spermatogenesis. J. Endocrinol..

[34] Yi W., Tang X.L., Zhou Y., Liu B., Shen L.J., Long C.L., Tao L., He D., Wu S. (2018). DEHP exposure destroys blood-testis barrier (BTB) integrity of immature testes through excessive ROS-mediated autophagy. Genes Dis..

[35] Shen Y.F., You Y.D., Zhu K., Li G.S., Huang X.P., Chen D., Yang F., Dong L., Li J., Yu X. (2023). The traditional Chinese medicine Qiangjing tablet prevents blood-testis barrier injury induced by CdCl through the PI3K/Akt/Rictor signaling pathway. Environ. Toxicol..

[36] Xiao X., Mruk D.D., Cheng C.Y. (2013). Intercellular adhesion molecules (ICAMs) and spermatogenesis. Hum. Reprod. Update.

[37] Kim S.E., Overholtzer M. (2013). Autophagy proteins regulate cell engulfment mechanisms that participate in cancer. Semin. Cancer Biol..

[38] Mossadegh-Keller N., Gentek R., Gimenez G., Bigot S., Mailfert S., Sieweke M.H. (2017). Developmental origin and maintenance of distinct testicular macrophage populations. J. Exp. Med..

[39] Rehman Z.U., Meng C., Sun Y., Safdar A., Pasha R.H., Munir M., Ding C. (2018). Oxidative stress in poultry: Lessons from the viral infections. Oxid. Med. Cell. Longev..

[40] Cecerska-Heryć E., Surowska O., Heryć R., Serwin N., Napiontek-Balińska S., Dołęgowska B. (2021). Are antioxidant enzymes essential markers in the diagnosis and monitoring of cancer patients—A review. Clin. Biochem..

[41] Fang F.C. (2011). Antimicrobial actions of reactive oxygen species. mBio.

[42] Ivanov A.V., Bartosch B., Isaguliants M.G. (2017). Oxidative stress in infection and consequent disease. Oxid. Med. Cell. Longev..

[43] Faraonio R. (2022). Oxidative stress and cell senescence process. Antioxidants.

[44] Loyo-Celis V., Sanghvi S.K., Raut S., Ponnalagu D., Singh H. (2024). Chloride intracellular channels regulate spermatogenesis. Biophys. J..

[45] Lin Q., Jin H.J., Zhang D., Gao L. (2020). DDX46 silencing inhibits cell proliferation by activating apoptosis and autophagy in cutaneous squamous cell carcinoma. Mol. Med. Rep..

[46] Yap Y.T., Li Y.H., Li W., Banerjee P., Zhang Z.B. (2021). ATP8a1, an IFT27 binding partner, is dispensable for spermatogenesis and male fertility. Mol. Reprod. Dev..

[47] Yin X.Q., Ma T., Han R.T., Ding J., Zhang H., Han X.D., Li D. (2018). MiR-301b-3p/3584-5p enhances low-dose mono-n-butyl phthalate (MBP) induced proliferation by targeting Rasd1 in Sertoli cells. Toxicol. In Vitro.

