# Supplementary material for: Alpha-Lipoic Acid Alleviates Lead-Induced Testicular Damage in Roosters by Reducing Oxidative Stress and Modulating Key Pathways
Source: Toxics. 2025 Apr 25;13(5):341. doi: 10.3390/toxics13050341 (PMC12116080; doi:10.3390/toxics13050341)
Supplement: Supplementary file 1 [file toxics-13-00341-s001.zip › toxics-3563785-supplementary.pdf]

Table S1. Ingredient composition and nutrient levels of the basal diet.

Ingredient (%)	Content
Corn	55.86
Soybean meal	19.10
Soybean oil	3.95
Wheat brain	15.12
NaCl	0.35
DL-methionine	0.15
Limestone	3.23
Dicalcium phosphate	1.52
Mineral premix	0.30
Vitamin premix	0.02
Choline chloride (50%)	0.40
Total	100
Calculation of nutrients and Pb content	
Metabolizable energy, MJ/kg	12.05
Crude protein	14.5
Calcium	2.5
Methionine	0.32
Lysine	0.7
Available phosphorus	0.5
Pb, mg/kg	≤ 5.0

Note: The following supplied per kg complete diet: Fe, 80 mg; Zn, 75 mg; Mn, 100 mg; Cu, 8 mg; I, 0.35 mg; Co, 0.2 mg; and Se, 0.15 mg. vitamin A, 12,500 IU; vitamin D3, 2500 IU; vitamin E, 80 mg; vitamin K, 2.65 mg; vitamin B1, 2 mg; vitamin B2, 6 mg; nicotinic acid, 50 mg; pantothenic acid, 12 mg; vitamin B6, 4 mg; folic acid, 1.25 mg; vitamin B12, 0.025 mg; folic acid, 1.25 mg; biotin, 0.0325 mg; niacin, 50mg. The Pb content in rooster feeds shall not exceed 5 mg/kg according to according to the Hygienical Standard for Feeds of China (GB 13078-2017).

Table S2. Criteria of spermatogenesis-scoring method.

Score	Criteria of Scoring Method
5	Complete spermatogenesis with complete tubules
4	Few or no spermatozoa but spermatids present
3	Few or no spermatids but spermatocytes present
2	Only spermatogonia present

1	No germ or Sertoli cells present

Table S3. DEGs identified in KEGG enrichment analysis.

Gene	$Log_2(FC)$	*p*-value	Regulation	Pathway
CON vs HPB				
CD8A	-1.2797	0.0012	Up	Cell adhesion molecules
MARCO	-1.3594	0.0382	Up	Phagosome
CDKN2A	1.1345	0.0020	Down	Cellular senescence
TBK1	-2.3220	0.0001	Up	virus infection disease
ITGA2	-1.0757	0.0293	Up	phagosome
HPB vs AHP				
CLDN19	-1.3649	0.0353	Up	Cell adhesion molecules
CD86	2.2568	1.99E-05	Down	Cell adhesion molecules
FAS	-1.8045	0.0023	Up	virus infection disease
MARCO	1.7893	0.0001	Down	phagosome
ITGA2	1.0241	0.0276	Down	phagosome
ITGA4	1.9124	0.0219	Down	ECM-receptor interaction
RASSF5	2.0282	0.0337	Down	Cellular senescence
F13A1	2.1117	1.35E-37	Down	Complement and coagulation cascades

Note: CD8A, CD8a molecule [Source: NCBI gene; Acc: 403158]; MARCO, macrophage receptor with collagenous structure [Source: NCBI gene; Acc: 395488]; *CDKN2A*, cyclin-dependent kinase inhibitor 2A (melanoma, p16, inhibits CDK4) [Source: NCBI gene; Acc: 395077]; *TBK1*, TANK binding kinase 1 [Source: NCBI gene; Acc: 417825]; *ITGA2*, integrin subunit alpha 2 [Source: NCBI gene; Acc: 100857227]; *CLDN19*, claudin 19 [Source: NCBI gene; Acc: 769245]; *CD86* , CD86 molecule [Source: NCBI gene; Acc: 427944]; *FAS* , Fas cell surface death receptor [Source: NCBI gene; Acc: 395274]; *ITGA4*, integrin subunit alpha 4 [Source: NCBI gene; Acc: 424121]; *RASSF5*, Ras association domain family member 5 [Source: NCBI gene; Acc: 419844]; *F13A1*, coagulation factor XIII A chain [Source: NCBI gene; Acc: 395420].

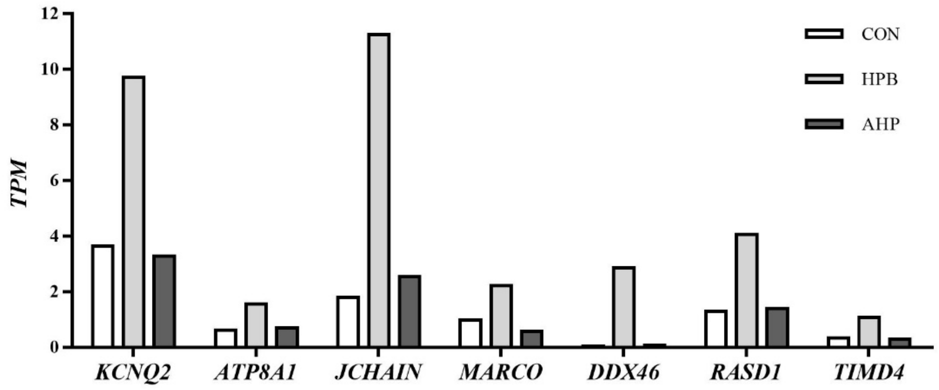

Figure S1. TPM of selected DEGs in rooster testes. TPM, transcripts per million. Groups: CON, roosters fed a basal diet; HPB, roosters fed a basal diet supplemented with 300 mg/kg $(CH_3OO)_2Pb$; AHP, roosters fed a basal diet supplemented with 300 mg/kg ALA and 300 mg/kg $(CH_3OO)_2Pb$. TPM values were calculated based on the number of transcripts, replacing the number of contigs with the number of transcripts. RSEM (RNA-Seq by Expectation-Maximization) software was used to perform quantitative analysis of gene and transcript expression levels. Data are presented as means (n = 3).
